# Analysis of the Influence of GMAW Process Parameters on the Properties and Microstructure of S32001 Steel

**DOI:** 10.3390/ma15186498

**Published:** 2022-09-19

**Authors:** Sandra Chacón-Fernández, Antonio Portolés García, Gerardo Romaní Labanda

**Affiliations:** Universidad Politécnica de Madrid (UPM), Escuela Técnica Superior de Ingenieros Industriales, c/José Gutiérrez Abascal, 2, 28006 Madrid, Spain

**Keywords:** welding, heat input, GMAW, duplex stainless steels (DSS), finite element model (FEM), diffusion

## Abstract

The properties of duplex steels can be seriously affected when welding processes are performed on them. Duplex S32001 is a recent development, so there is very little published information on its behavior after a welding process. For this reason, the present article determines how the main welding parameters of the GMAW process influence its mechanical and microstructural properties. From this study, it was determined that the parameter W/m^3^ best defines the phase balance in the bead due to the values involved. In the HAZ, this parameter is the t_12/8_. The welding parameters that are used determine the bead dimensions and geometry. These dimensions induce differences in the distribution of heat in the material. For this reason, the thermal cycles undergone by the material vary and, consequently, in the phase balance obtained. A theoretical study of the chemical composition in the bead, through dilution, and in the HAZ, through diffusion, was carried out. A mathematical model was developed that allows the variation of the composition in the HAZ that induces diffusion to be determined.

## 1. Introduction

Duplex stainless steels (DSS) are Fe-Cr-Ni alloys containing mainly two phases in large quantities, austenite and ferrite, whose ideal balance is 50/50%. The duplex steel will have better properties, as the alloys are closer to the ideal balance [1].

This research was carried out on duplex steel S32001, newly created, whose chromium content ranges between 19.50% and 21.50%. It has good toughness, an elastic limit twice that of AISI type 304 and 316 stainless steels, good resistance to general corrosion and stress corrosion cracking, and good formability. The S32001 duplex has fewer alloying elements in its composition than the duplexes belonging to the standard group, so its resistance to corrosion is lower. These properties, in addition to the physical and chemical ones, are given by the composition and the thermal history, which determine the microstructure and the phase balance of the alloys. The duplex S32001, due to its lower cost, is gaining importance in the manufacture of process and storage tanks, ducts and pipes, structural applications, catwalks, strapping, clamps, cable trays, and equipment for the food industry in aggressive environments [2,3,4,5].

There is very little published information on S32001 steel and its behavior after a welding process. For this reason, it is important to analyze the influence of the welding parameters on these steels because the thermal cycles undergone by the material modify the microstructure and properties. These modifications make it possible to distinguish three differentiated zones in the material: the weld bead, the heat affected zone (HAZ), and the base metal (BM) [6,7,8,9]. For ferrite to transform into austenite, a certain amount of time must elapse: if the cooling rate is too high, the transformation will not occur and the phase relationship will not be as desired [10]. 

The GMAW (gas metal arc welding) welding process was selected due to its wide use in the industry. This process can be executed both semi-automatically and fully robotized.

The energy input in the welding process is another very important parameter in influencing the phase relationship and properties of the welded zone. The heat input is the amount of energy applied per unit weld length, (kJ/mm). In the GMAW process where the heat source is an electric arc, the formula for the heat input is given by:

(1) $$H=\rho \;\frac{UI}{v}\;10^{-3}$$

where U is the voltage (V), I is the current (A), v is the wire feed speed (mm/s), and ρ is the process performance parameter (0.8 for GMAW welding, according to the UNE-EN 1011-1 standard) [11,12].

In this paper, the influence of the main GMAW robotic welding parameters on the microstructures of the weld bead and HAZ is analyzed. The microstructure is studied by optical microscopy, while the influence of the different parameters and thermal cycles is analyzed with the help of finite element models [9,13]. The mechanical properties are studied by tensile testing and hardness chains. The diffusion in the HAZ of the main alloys is analyzed according to the difference in chemical composition between the bead (obtained from dilution) and the base metal. In this way, it is possible to determine their influence on the mechanical properties of the welded joint.

In view of the results obtained, it was determined that the parameter W/m^3^ best defines the phase balance in the bead due to the values involved. In the HAZ, this parameter is the t_12/8_. Regarding the dimensions and geometry obtained in the welding bead, the behavior is directly related to the main welding parameters studied. These dimensions induce differences in the distribution of heat in the material and, therefore, in the thermal cycles undergone by the material and, consequently, in the phase balance obtained. A small statistical study using the correlation coefficient (R^2^) made it possible to reinforce the conclusions obtained.

## 2. Materials and Methods

### 2.1. Materials

The base metal used was lean duplex stainless steel UNS S32001 (LDSS, Acerinox, Madrid, Spain), with coupons of dimensions 200 mm × 150 mm × 3 mm (without bevel), and the filler material was a 1.2 mm diameter wire of duplex stainless steel ER2209. Table 1 shows the chemical composition of the base metal and the filler metal used (according to the manufacturer’s data) [14]. The mechanical properties measured for the as-received material were 753.7 N/mm^2^ for the tensile strength and 234 HV for the hardness.

### 2.2. Methods

#### 2.2.1. Welding

**Figure 1:**
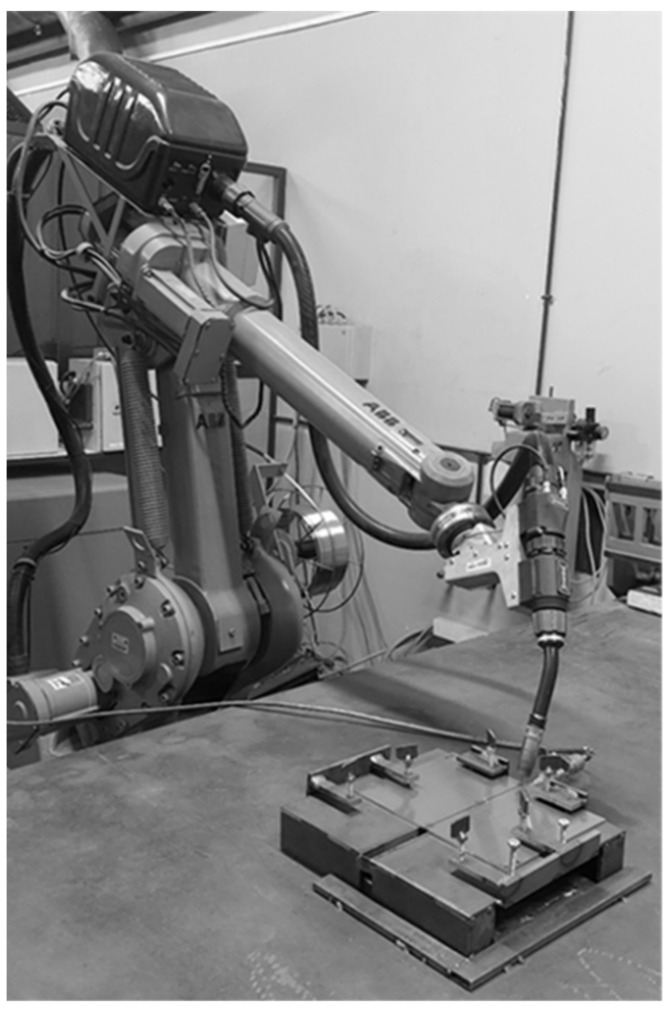
Welding robot and anchoring system.

The welds were made using the ABB IRB 1400 robot (AristoRob 500 power source) with the pulsed arc method in a single pass because the selected thickness allows it, thus avoiding the influence of other factors such as the temperature between passes and the sequence of passes. The welding was carried out on two flat sheets without a bevel with a gap between them of 1.5 mm. A gas channel anchoring system was used to prevent the coupons from moving during welding and to apply a shielding gas in the root zone of the weld (Figure 1). Argon with 2% CO_2_, which helps to improve the arc stability, was used as a shielding gas, with flow rates of 14 L/min in the bead face zone and 1 L/min in the root zone (as a backing). 

The influence of the main parameters affecting the welding was studied: current, welding speed, voltage, and wire feed speed. Table 2 shows the study parameters, as well as the actual current and voltage values used during the welding of the test coupons and the heat input. The variation of the parameters is limited so as not to cause defects in the welding coupons. This energy can be classified as low heat input (coupon No. 4), medium heat input (coupon No. 1), and high heat input (coupon No. 2).

The extraction of the samples from the welding coupons for their characterization study was carried out following the recommendations of the UNE-EN ISO 15614-1:2018 standard [15].

#### 2.2.2. Microstructural Characterization and Grain Size

The samples were hot-mounted in a resin for easy handling. They were first water-sanded using 240 to 4000 grit sandpaper and then polished with a 1 µm monocrystalline diamond suspension. A final cleaning of the samples was carried out using an ultrasonic water bath. The polished surface was chemically etched by immersion for 15 s with Beraha I reagent (20 mL HCl, 100 mL H_2_O, 1 g K_2_S_2_O_5_, 2.4 g NH_4_FHF) [16,17].

The α/γ phase ratio of each sample was obtained by image analysis with Perfect Image^®^ software (Clara Vision, Bièvres, France).

For the determination of the grain size, the UNE-EN ISO 643:2020 standard [18] was followed with the Heyn measurement method. In parallel, the comparison method was used to check the reliability of the measurements. The images were processed using Grani^®^ software (Clara Vision), which allowed the application of these methods. The grain size was determined only in the HAZ, as this is the zone where the grain size increases and can affect the material properties.

#### 2.2.3. Vickers Hardness, Tensile Testing, and Weld Dimensions

To obtain the hardness, the recommendations of the UNE-EN ISO 6507-1:2018 standard [19] were followed. The test was performed by means of a hardness chain, and the measurements were taken at a 1 mm depth from the surface. The hardness was taken in the three zones of the weld (the bead, the HAZ, and the BM). Three measurements were taken for each zone. The test was carried out at room temperature of about 25 °C using the Vickers method, with a load of 1 kg and a 30 s penetration time.

To determine the dimensions of the weld bead, the recommendations of UNE-EN ISO 5817:2014 [20] were followed, obtaining the dimensions in the face bead of the excess weld metal and width and in the root zone of the excess penetration and width. The measurements were performed with a Nikon V-12A (Tokyo, Japan) profile projector.

For tensile testing, the UNE-EN ISO 6892-1:2017 [21] standard was followed at room temperature with the MTS 810 equipment.

#### 2.2.4. Finite Element Modeling

Abaqus^®^ V 6.14 (SIMULIA, Dassault Systemes, Velizy-Villacoublay, France) specific software of proven reliability was used to create the finite element model. Half of the part was modeled three-dimensionally (because the part is symmetrical with respect to the longitudinal axis of the bead) on which the heat input during the welding process was simulated. The following temperature-varying material properties were used to build the model: density, thermal conductivity, specific heat, and latent heat of fusion [22,23]. The simulation of the model was performed according to the previous experience of some of the authors [24,25,26]. The elements that compose the bead (real dimensions) are the heat generators. The initial temperature of the room in the modeling was considered to be 20 °C. For the boundary conditions, heat transfer by conduction through the material and by convection and radiation through the surfaces was assumed.

The study focused on simulating the thermal cycle undergone by each of the three zones of the weld (bead, HAZ, and BM) and determining the cooling rate and the maximum temperature reached of each zone. The thermal model was made following the most common considerations accepted by other authors [10,27,28].

**Figure 2:**
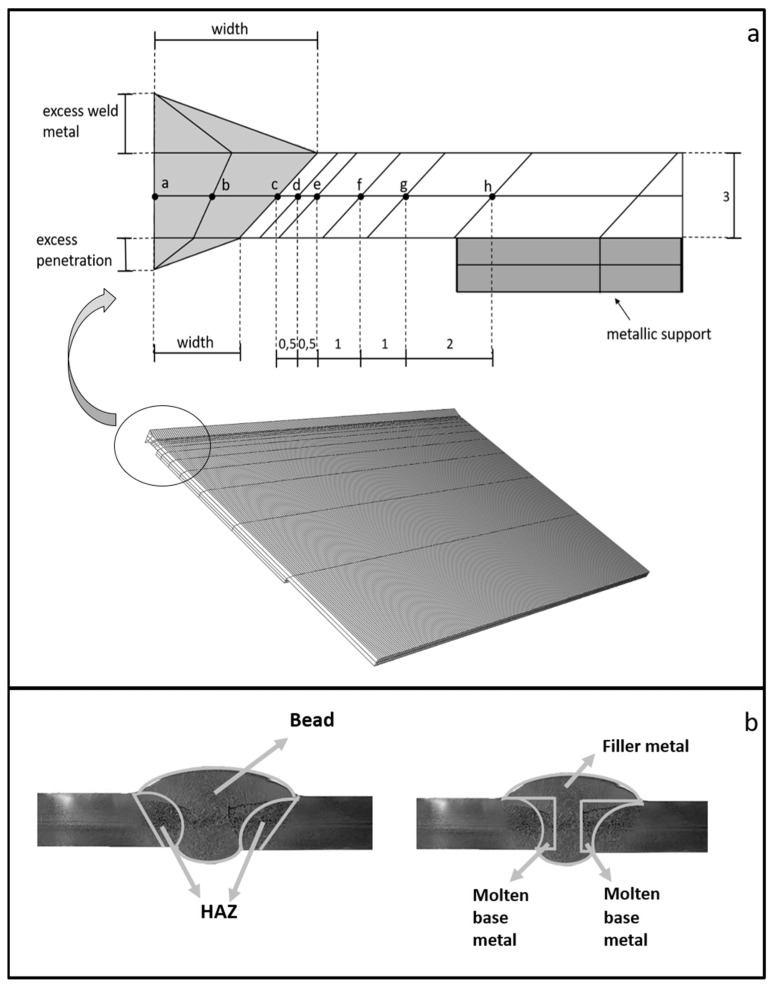
(**a**) Mesh used in FEM, (**b**) diagram of the zones studied for the dilution and diffusion of the alloys.

Figure 2a shows the model used for the finite element simulation. For greater fidelity, the metal support on which the part is supported in reality was also modeled. Point a (located in the central zone) is the node chosen to represent the weld bead zone. Point d (at a distance of 0.5 mm from the HAZ interface) is the node chosen to represent the HAZ. 

The results obtained with the finite element simulation are: the thermal cycle undergone by each node, the t_12/8_ (time elapsed between the temperatures from 1200 to 800 °C), and the heat distribution in the material.

#### 2.2.5. Alloy Dilution and Diffusion

To complete the study of the influence of the welding parameters on the bead and HAZ, a study of the dilution obtained in the molten zone and the diffusion occurring in the HAZ and in the bead was carried out. These data allow us to determine the diffusion rate of the main alloys of the material (Cr, Ni, Mn, and Mo), as well as the theoretical composition obtained in each of the nodes of the mesh of Figure 2a (nodes a–h). These results determine its hardness, mechanical properties, and corrosion resistance. The data obtained from the thermal cycling of each node of the finite element models were used for this study.

The dilution was calculated by measuring the area corresponding to the base metal in the weld bead.

(2) $$Dilution\;\left(\%\right)=\frac{B}{A+B}\;100$$

where B is the amount of base metal that is part of the bead and A is the amount of filler metal [29].

Figure 2b shows, as an example, the different zones of the weld bead and how the amount of base metal and molten filler metal forming the weld bead was measured on one of the coupons. 

The composition is considered to be homogeneous (zone between nodes a and c in Figure 2a) in the molten zone (weld bead), so the diffusion study of the bead was performed starting from node c. The diffusion study was performed between nodes c and h. Nodes following node h were not studied because the temperatures reached are below 500 °C. The temperature range studied was 1370–800 °C (the solidus line of S32001 [23] is located at 1370 °C).

For the calculation of the diffusion velocity, the Arrhenius formula was used [30]:

(3) D=A e−QRT

where A is the frequency factor (cm^2^/s), Q is the activation energy (kJ/mol), R is the universal gas constant (8.3143 J/mol K), and T is the temperature (in K). 

## 3. Results and Discussion

### 3.1. Weld Dimensions

Table 3 shows the dimensions obtained with the profile projector of all the samples, both at the face and at the root.

**Figure 3:**
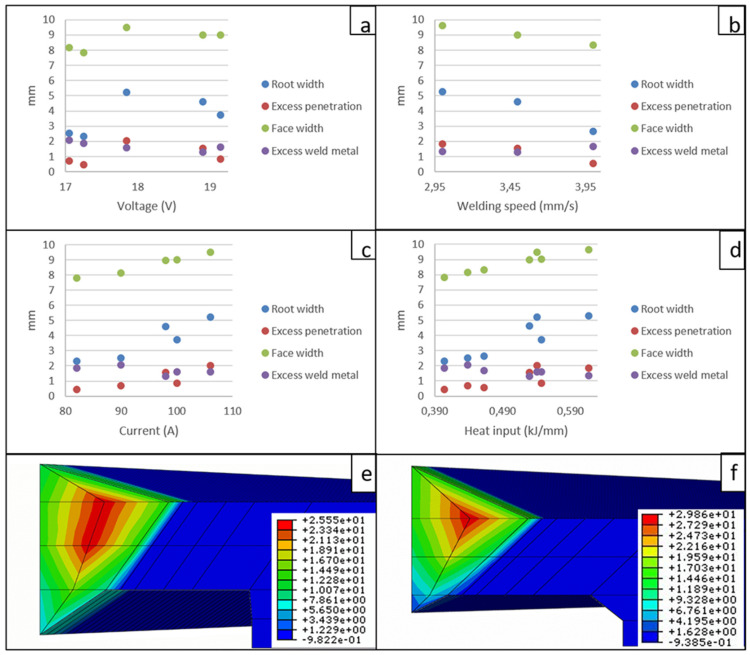
Influence of the parameters on the weld bead dimensions, (**a**) voltage, (**b**) welding speed, (**c**) current, (**d**) heat input, (**e**) heat flow distribution on coupon 1 (lower excess weld metal), (**f**) heat flow distribution on coupon 6 (higher excess weld metal).

Figure 3 shows the influence of the different parameters on the weld dimensions. Figure 3a shows the influence of voltage; an increase of this parameter leads to a longer arc length, which favors a larger bead width (with a correlation coefficient value of R^2^ = 0.994), decreasing the weld metal, excess unless the welding feed rates are higher. Figure 3b shows the influence of welding speed. An increase in speed implies that the time needed for the base metal to melt is insufficient, so the excess weld metal is higher (with R^2^ = 0.923). Similarly, a lower excess penetration (R^2^ = 0.974) and a smaller face width (R^2^ = 0.999) and weld bead root (R^2^ = 0.899) are obtained. Figure 3c,d show the influence of current and heat input, respectively. In both cases, the behavior is similar. The increase in current is directly related to the increase in filler metal, which causes the heat input in the zone to be higher. For this reason, the width of the bead face increases (R^2^ = 0.919) and excess penetration is favored (R^2^ = 0.956). A lower current input leads to a lower heat input, which can lead to a higher excess weld metal (R^2^ = 0.888) and, consequently, to bead dimensions being out of norm. The dimensions of the bead directly influence the heat flow in the welded joint (Figure 3e,f) because, depending on the geometry, the heat distribution varies and influences the cooling rate. In Figure 3e, it can be seen that having a smaller weld metal excess favors a more uniform heat flow toward the base metal throughout the thickness.

The statistical result of the correlation coefficient R^2^ allows the results obtained in relation to the main welding parameters and their direct influence on the dimensions of the weld to be corroborated.

### 3.2. Microstructural Characterization and Grain Size

Welding processes produce very high temperatures and rapid cooling rates in the weld bead. As the distance to the molten zone increases, the maximum temperature is reached and the cooling rate decreases, which causes the appearance of three zones with different microstructures in the welded joint (weld bead, HAZ, and BM).

**Figure 4:**
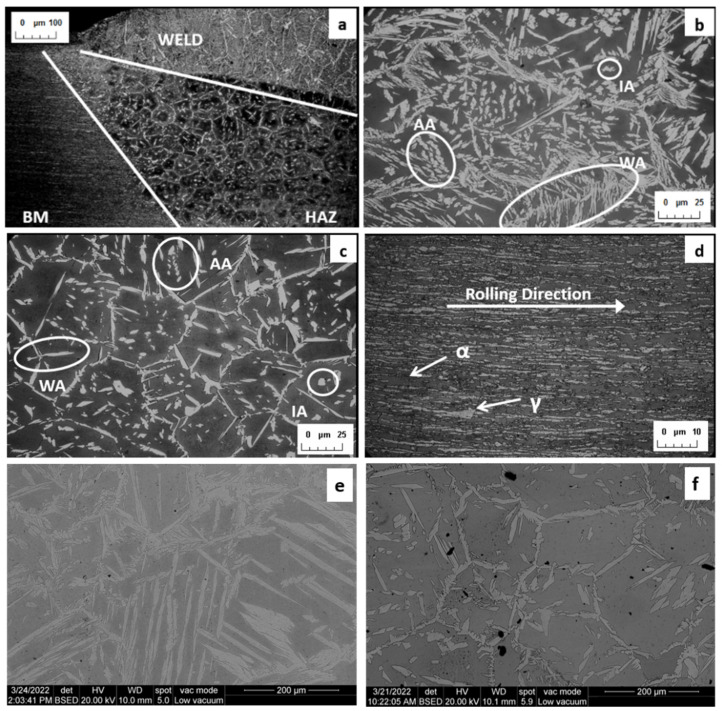
Microstructure obtained on weld coupon 3. (**a**) The three types of microstructure are shown: bead, HAZ, and BM (base metal), (**b**) weld bead, (**c**) HAZ, (**d**) base metal, SEM images (**e**) of the weld bead, (**f**) of the HAZ.

Figure 4 shows the microstructure corresponding to coupon 3. The microstructure of the rest of the coupons is similar. The dark zone is the ferrite phase and the illuminated zone is the austenite phase. Figure 4a,e,f are merely illustrative. Figure 4a shows an overview of the three zones of the weld: the bead, the HAZ, and the BM. It can be seen that due to the very high temperature heating, recrystallization and an increase in grain size occur in the HAZ. Figure 4e,f correspond to SEM images of the bead and HAZ, respectively. Figure 4d shows the base metal; it is an oriented structure as a result of a cold deformation process. It reveals the rolling direction and the structure in the form of alternating layers of ferrite and austenite. Figure 4b,c correspond to the weld bead and the HAZ, respectively. In both, a similar microstructure can be observed with austenite in different morphologies: Widmanstätten austenite needles (WA) formed by rapid cooling to room temperature of the weld, allotropic austenite (AA), and intragranular secondary austenite (IA). In the HAZ, the grain cell morphology appears and the austenite surrounds the ferrite grains. This is a favorable situation because the austenite prevents cracks from propagating in the event of crack formation due to the higher toughness of the austenite. 

It has been observed that the grain size in the HAZ grows in a smaller proportion as the excess weld metal increases as a consequence of an increase in the voltage applied in the process. The heat distribution in the welded zone changes, Figure 3e,f, as a consequence of a shorter time at an elevated temperature and an increase in the cooling rate.

### 3.3. Alloy Dilution and Diffusion

Table 4 shows the results of the chemical composition of the study alloys in the bead. These results were obtained from the chemical composition of the filler metal, the base metal, and the dilution value.

The following considerations were taken into account to calculate the diffusion rate:

- The diffusion rate of alloying agents is favored at elevated temperatures [31].
- The ideal situation of only the ferritic matrix (α-Fe) existing between 1370 and 1201 °C was assumed.
- Two temperature ranges 1370–1201 °C and 1200–800 °C were studied.
- Between 1200 and 800 °C, the transformation of ferrite to austenite occurs linearly, reaching a ferrite percentage similar to that of the base metal, provided that at least 10 s elapse in this cooling [9].
- The calculated results of the diffusion velocity below 800 °C (according to Equation (3)) are of the order of 10^−7^ mm/s, so they can be disregarded. As a consequence, from node g (located 3 mm away from the bead-HAZ interface, node c) onwards, the base metal composition was achieved.
- The variation of the composition from the bead to the base metal occurs between nodes c and g because this is where the highest temperature range occurs.

**Figure 5:**
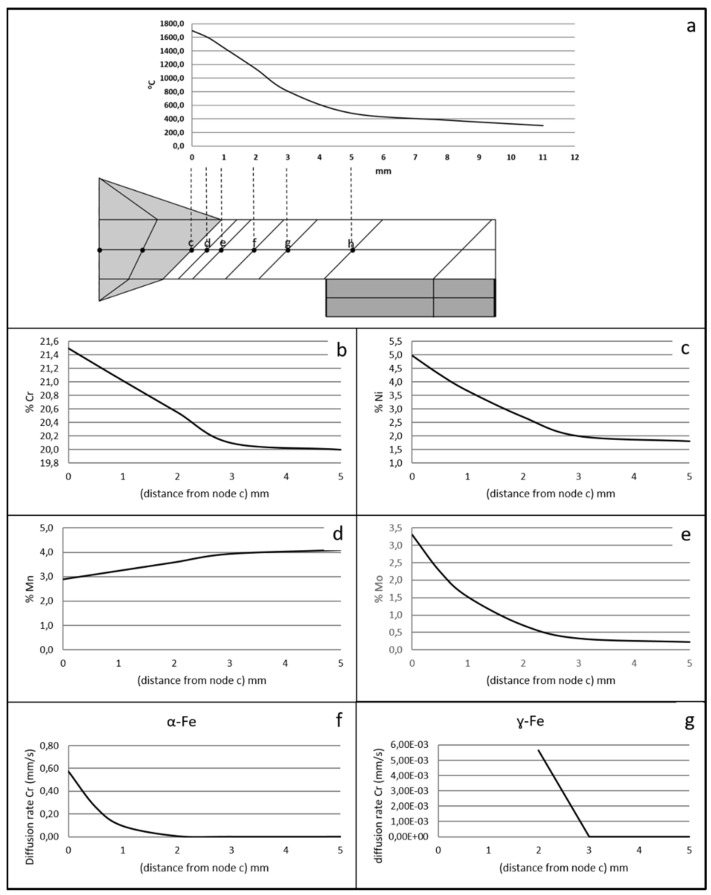
(**a**) Thermal cycling of the study nodes, Variation of the composition of the study nodes: (**b**) chromium, (**c**) nickel, (**d**) manganese, (**e**) molybdenum, (**f**) diffusion rate of chromium in α-Fe, (**g**) diffusion rate of chromium in ɣ-Fe.

From the temperature distribution obtained in the mesh nodes in one of the coupons (it was determined that in the rest, they evolve in the same way, Figure 5a), a mathematical model was developed to determine the function that relates the chemical composition variation in the mesh nodes with their distance from node c. 

The model made it possible to obtain the two equations that adjust to this behavior, depending on the theoretical dilution composition obtained at node c and the composition of the base metal. Thus, the elements Cr, Ni, and Mo follow the equation:

(4) E=Ebe−adEbm

and Mn follows the equation:

(5) $$E=d\ln\frac{E_{bm}}{a}+E_{b}$$

where E is the percentage of the element desired, $E_{b}$ is the percentage of the element in the dilution (weld bead), d is the distance (mm) from node c for which the composition is being calculated, $E_{bm}$ is the percentage of the element in the base metal, and a is a curvature adjustment coefficient, which has been calculated considering that diffusion below 800 °C is insignificant.

Figure 5b–e show the graphical representation of the evolution of the composition at the study nodes.

Once the composition at each node was determined, the diffusion rate of each element at each node was calculated. As an example, the result obtained for chromium in both α-Fe and ɣ-Fe is shown in Figure 5f,g. We proceeded to determine the equation governing this behavior, obtaining:

(6) vd=vbe−advbm

where $v_{b}$ is the diffusion velocity at node c, $v_{bm}$ is the diffusion velocity in the base metal, d is the distance to node c (mm), and a is a curvature adjustment coefficient.

### 3.4. Phase Balance and Finite Element Modeling

A reliability study (Cronbach’s alpha) [32] was carried out with the measurements obtained in the phase balance of the two study zones, the bead, and the HAZ. For this purpose, six measurements were made in the strand and in the HAZ. At the end of the study, the results were between 0.857 and 0.997, which indicates a high reliability.

To analyze the parameters that influence the phase balance of each zone, points a and d in Figure 2a were taken as a reference. 

**Figure 6:**
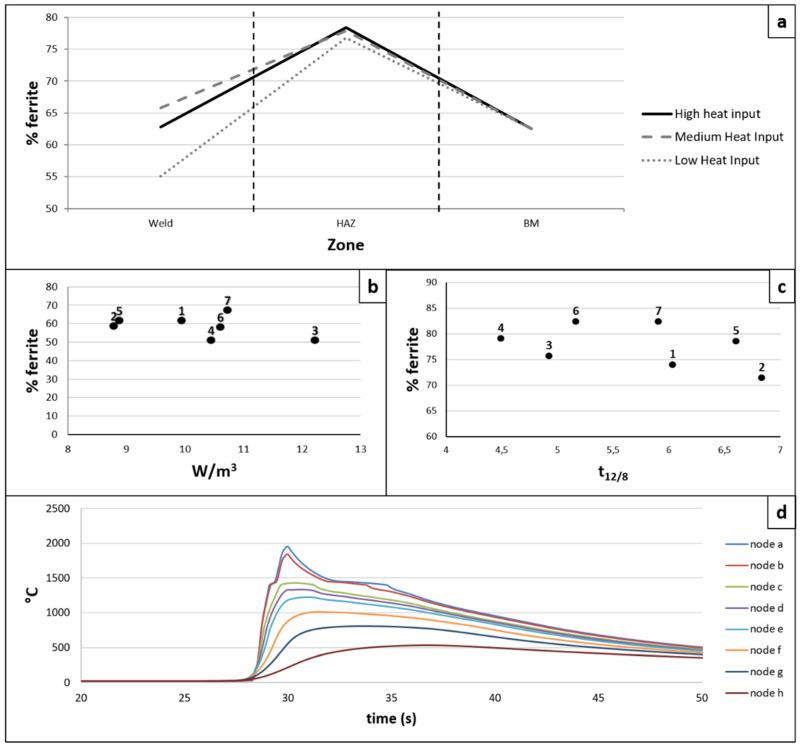
(**a**) Amount of ferrite obtained for the coupons with the highest, medium, and lowest thermal input, depending on the weld zone, (**b**) % ferrite in the weld bead vs. W/m^3^, (**c**) % ferrite vs. time t_12/8_, (**d**) thermal cycle suffered by the nodes in weld coupon 2.

Figure 6a shows the amount of ferrite obtained from the phase balance of each weld coupon. For the graphical representation, the samples corresponding to the coupons subjected to high, medium, and low heat input (according to the classification indicated above) were selected. 

In the bead zone, it was determined that the most influential parameters are: the maximum temperature reached, the geometry of the bead section, and the cooling rate. All of them are related to the power transferred per unit volume (W/m^3^) [11]. Figure 6b shows the influence of this transferred power density (W/m^3^) on the amount of ferrite found. A high value of W/m^3^ induces a slower cooling rate, which can also favor the diffusion and absorption of nitrogen in the material, favoring the transformation of ferrite into austenite [33,34]. The W/m^3^ is also the parameter with the greatest influence on the dilution obtained.

In the HAZ, the most influential parameter is the time t_12/8_. In this temperature range, the transformation of ferrite into austenite takes place, so the longer it remains in this temperature range, the more ferrite is transformed and the phase balance is more balanced [11]. Figure 6c shows the result obtained.

Figure 6d shows, as an example, the thermal cycling of welding coupon 2 for the corresponding bead and HAZ nodes at 1.5 mm from the surface. 

### 3.5. Vickers Hardness and Tensile Testing

**Figure 7:**
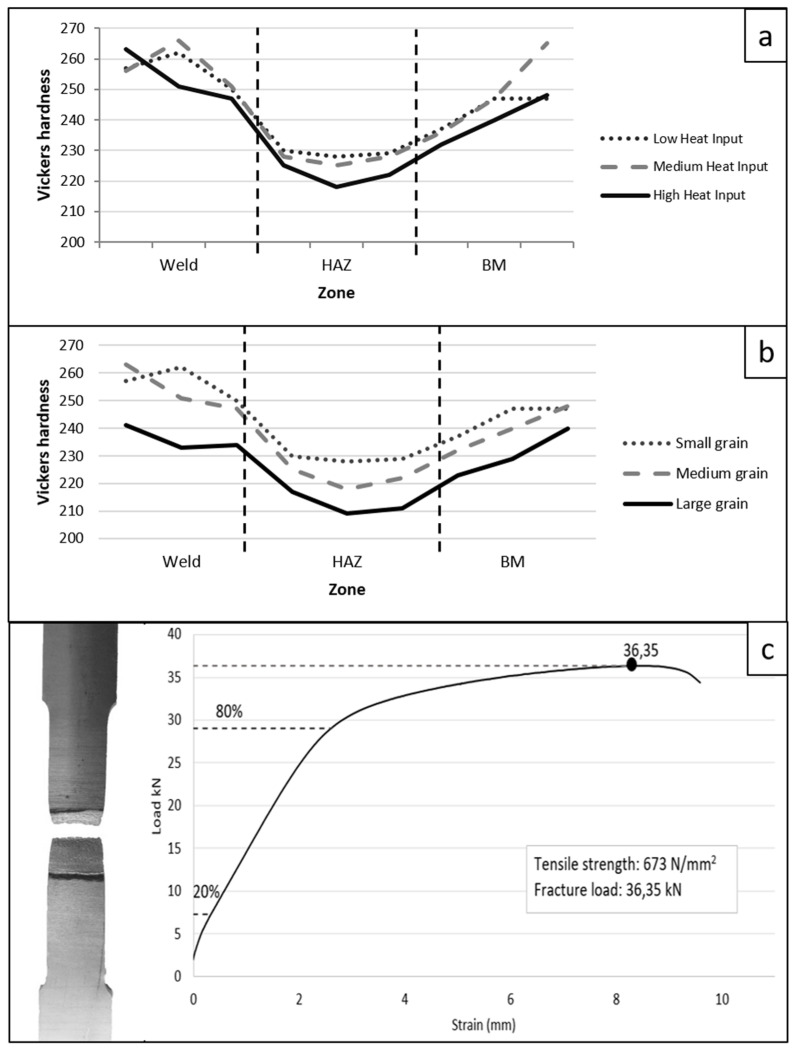
(**a**) Vickers hardness as a function of the heat input of material S32001. (**b**) Vickers hardness as a function of grain size, (**c**) tensile testing for coupon 7.

Figure 7a shows the hardness obtained for material S32001. It can be seen that the filler metal, which has a composition that provides better mechanical properties, increases the hardness in the bead zone. In the HAZ, the decrease in hardness is influenced by the recrystallization undergone, the increase in grain size, and the phase relationship. It was also observed that samples with higher nickel and chromium content (obtained from the diffusion study) show a higher hardness in the bead and HAZ [35]. The highest hardness is observed in the coupons with lower heat input due to the fact that the temperatures reached are lower and the cooling speed is higher.

Figure 7b shows the variation of the hardness as a function of grain size; the smaller the grain size, the higher the hardness of the zone. The small grain size corresponds to coupon 4, the medium size to coupon 2, and the large size to coupon 6.

Figure 7c shows, as an example, an image of coupon 7 and the results of the tensile testing. In all cases, a ductile fracture was obtained. All the samples were broken at 90° with respect to their longitudinal axis through the zone of the HAZ, indicating that this zone is the weakest due to the variation of the phase balance and increase in the grain size. Table 5 shows the results obtained in the tensile testing for tensile strength, fracture load, and joint efficiency in all weld coupons.

To calculate the Joint efficiency, the following equation was used:

(7) $$Joint\;efficiency=\frac{\mathrm{tensile}\;\mathrm{strength}\;\mathrm{cup}ón}{\mathrm{tensile}\;\mathrm{strength}\;\mathrm{BM}}*100$$

All tensile strength values are within those specified by the supplier in the annealed state (660–900 N/mm^2^). For this reason and because the weakest zone is the HAZ, a less alloyed filler metal could be used as long as it ensures the minimum mechanical properties required of the base metal. 

The lowest values of hardness and tensile strength are obtained in coupons with larger grain size.

## 4. Conclusions

In order to analyze the effect of GMAW welding of DSS S32001, the influence of different robotic welding parameters on the microstructure and properties obtained in the different welding zones was studied.

To complement the information on the thermal cycle that the steel undergoes during welding, a finite element modeling was also carried out to analyze in detail the parameters of influence in each of these welding zones. Based on the information obtained from the finite element model and the geometry of the weld beads, the dilution in the bead zone was determined and a mathematical model for the diffusion of the main alloying agents was developed.

The conclusions drawn from the study are summarized below:

- The microstructure obtained in the bead and in the HAZ is similar, obtaining needle-shaped austenite (Widmanstätten), allotropic austenite, and intragranular austenite, although the amount of needles found in the HAZ is lower.
- Compared to the heat input, it was observed that the parameter W/m^3^ better explains the phase balance behavior in the weld bead due to the parameters related to this value.
- The distribution of the heat flow toward the base metal is conditioned by the dimensions and geometry of the bead, which influences the thermal cycles undergone by the material and, consequently, the phase balance obtained.
- To determine the geometry of the bead, it is necessary to study the influence of the parameters: voltage, current, and welding speed, individually and jointly by means of the heat input value.
- The mathematical model developed for diffusion allows the chemical composition at each node of the mesh to be obtained and, consequently, the behavior of the material to be predicted. In this way, the characteristics of the welded joint can be improved to suit the required needs.
- The t_12/8_ determines the cooling rate in the HAZ and, therefore, the phase balance. So, by controlling the cooling rate (which is conditioned by the heat input and bead geometry), a more balanced phase balance can be obtained in this zone.
- The HAZ is the most weakened zone during the welding process due to the increased grain size and higher ferrite content, which leads to a loss of hardness and tensile strength.

The results obtained with this study allow us to determine the welding parameters to be able to qualify procedures for ducts and pipes, structural applications, and walkways.

## Figures and Tables

**Table 1:** Chemical composition of duplex stainless steel (DSS) UNS S32001 and filler metal ER2209 (wt %).

Material	C	Mn	Si	P	S	Cr	Ni	Mo	N
S32001	0.016	4.1	0.56	0.026	0.004	20	1.8	0.22	0.14
ER2209	0.01	1.45	0.45	0.015	0.015	23.2	8.6	3.3	0.17

**Table 2:** Welding parameters for GMAW on duplex stainless steel S32001.

Coupon	Welding Speed (mm/s)	Wire Feed Speed (m/min)	Current (A)	Voltage (V)	Heat Input (kJ/mm)
1	3.5	2.75	98	18.9	0.423
2	3	2.75	98	18.9	0.494
3	4	2.75	98	18.9	0.370
4	3.5	2.4	82	17.25	0.323
5	3.5	3.25	106	17.85	0.432
6	3.5	2.75	90	17.05	0.351
7	3.5	2.75	100	19.15	0.438

**Table 3:** Dimensions of all welds.

Coupon	Root Dimensions (mm)		Face Dimensions (mm)	
	Width	Excess Penetration	Width	Excess Weld Metal
1	4.6	1.5	8.9	1.3
2	5.3	1.8	9.6	1.4
3	2.7	0.5	8.3	1.7
4	2.3	0.4	7.8	1.9
5	5.2	2.1	9.5	1.6
6	2.5	0.7	8.2	2.1
7	3.7	0.9	9.1	1.6

**Table 4:** Composition after dilution at node c.

Coupon	BM Dilution %	Cr	Ni	Mn	Mo
1	53.3	21.5	5.0	2.9	1.7
2	53.0	21.5	5.0	2.9	1.7
3	49.5	21.6	5.2	2.8	1.8
4	46.6	21.7	5.4	2.7	1.9
5	50.3	21.6	5.2	2.8	1.8
6	45.5	21.7	5.5	2.7	1.9
7	52.2	21.5	5.1	2.8	1.7

**Table 5:** Values obtained in the tensile testing for tensile strength, fracture load, and joint efficiency for welding coupons.

Coupon	Tensile Strength (N/mm^2^)	Fracture Load (kN)	Joint Efficiency (%)
1	690	34.74	91.55
2	698.5	34.86	92.68
3	687	36.75	91.2
4	689.4	35.28	91.47
5	672	34.32	89.16
6	688.1	34.09	91.3
7	673	36.35	89.29

## Data Availability

Not applicable.
